# Automated machine learning for the identification of asymptomatic COVID-19 carriers based on chest CT images

**DOI:** 10.1186/s12880-024-01211-w

**Published:** 2024-02-27

**Authors:** Minyue Yin, Chao Xu, Jinzhou Zhu, Yuhan Xue, Yijia Zhou, Yu He, Jiaxi Lin, Lu Liu, Jingwen Gao, Xiaolin Liu, Dan Shen, Cuiping Fu

**Affiliations:** 1 Department of Gastroenterology, The First Affiliated Hospital of Soochow University, 215006 Suzhou, Jiangsu China; 2 Department of Respiratory Medicine, The First Affiliated Hospital of Soochow University, 215006 Suzhou, Jiangsu China; 3 Medical School, Soochow University, 215006 Suzhou, Jiangsu China; 4 The 23th ward, Yangzhou Third People’s Hospital, 225000 Yangzhou, Jiangsu China; 5 Suzhou Clinical Center of Digestive Diseases, 215006 Suzhou, Jiangsu China; 6 Department of Radiotherapy, The First Affiliated Hospital of Soochow University, 215006 Suzhou, Jiangsu China

**Keywords:** Automated machine learning, COVID-19, Asymptomatic, Prediction model

## Abstract

**Background:**

Asymptomatic COVID-19 carriers with normal chest computed tomography (CT) scans have perpetuated the ongoing pandemic of this disease. This retrospective study aimed to use automated machine learning (AutoML) to develop a prediction model based on CT characteristics for the identification of asymptomatic carriers.

**Methods:**

Asymptomatic carriers were from Yangzhou Third People’s Hospital from August 1st, 2020, to March 31st, 2021, and the control group included a healthy population from a nonepizootic area with two negative RT‒PCR results within 48 h. All CT images were preprocessed using MATLAB. Model development and validation were conducted in R with the H2O package. The models were built based on six algorithms, e.g., random forest and deep neural network (DNN), and a training set (*n* = 691). The models were improved by automatically adjusting hyperparameters for an internal validation set (*n* = 306). The performance of the obtained models was evaluated based on a dataset from Suzhou (*n* = 178) using the area under the curve (AUC), accuracy, sensitivity, specificity, positive predictive value (PPV), negative predictive value (NPV) and F1 score.

**Results:**

A total of 1,175 images were preprocessed with high stability. Six models were developed, and the performance of the DNN model ranked first, with an AUC value of 0.898 for the test set. The sensitivity, specificity, PPV, NPV, F1 score and accuracy of the DNN model were 0.820, 0.854, 0.849, 0.826, 0.834 and 0.837, respectively. A plot of a local interpretable model-agnostic explanation demonstrated how different variables worked in identifying asymptomatic carriers.

**Conclusions:**

Our study demonstrates that AutoML models based on CT images can be used to identify asymptomatic carriers. The most promising model for clinical implementation is the DNN-algorithm-based model.

## Introduction

Coronaviruses are widely distributed pathogens in humans and other animals and can cause enteric, neurologic, and respiratory illnesses ranging from the common cold to fatal infections [1]. Timely and accurate diagnosis of COVID-19 is of utmost importance for the prompt treatment of patients and their isolation. The diagnosis is confirmed by reverse-transcription polymerase chain reaction (RT‒PCR). Typical manifestations of COVID-19 pneumonia are para-pleural ground-glass opacity (GGO), interlobular septal thickening, central consolidation of the focus and banded atelectasis [1, 2]. The National Health Commission of the People’s Republic of China initially proposed screening based only on clinical and chest computed tomography (CT) findings. However, recently, asymptomatic carriers have perpetuated the ongoing pandemic of this viral disease [3–5]. It is difficult to timely and accurately reflect the internal viral load on the basis of throat swab samples. Negative RT‒PCR results for throat swab samples are not the gold standard of exclusion. Transmission of the novel COVID-19 from an asymptomatic carrier with normal CT findings has been reported. The CT images of the asymptomatic patients are initially judged as normal by radiologists. However, some asymptomatic infections develop into pneumonia in later weeks [6]. The rapid person-to-person transmission among asymptomatic carriers is difficult to discover in the clinic. As the full liberalization of COVID-19, early recognition of COVID-19 pneumonia would help determine the degree of the disease and promote early treatment, thereby preventing viral pneumonia. Thus, it is not enough for clinicians alone to assess the CT characteristics of asymptomatic patients. Applications of artificial intelligence (AI) will help identify CT characteristics specific to asymptomatic patients.

AI is rapidly entering the medical domain and is being used for a wide range of health care and research purposes, including disease detection [7], empirical therapy selection [8], and drug discovery [9]. The complexity and growing volume of health care data indicate that AI techniques will increasingly be applied in almost every medical field in the upcoming years. Recent studies have demonstrated that AI may prove extremely helpful in the medical imaging domain due to its high capability for identifying specific disease patterns. Studies have proposed several machine learning models that can accurately predict COVID-19 disease severity [10–12]. A comprehensive bibliometric analysis was performed to summarize all accessible techniques for detecting, classifying, monitoring and locating COVID-19 patients, including AI, big data and smart applications [13]. They concluded that AI-assisted CT was better at diagnosing COVID-19 pneumonia due to its high precision and low false-negative rates. However, models have rarely been built to separate asymptomatic from healthy individuals. This study was designed to (1) develop predictive models by using automated machine learning (AutoML), characterized by automated hyperparameter adjustment, and (2) choose the best performing machine learning model based on CT radiomic features for the identification of asymptomatic COVID-19 patients.

Machine learning models have often been criticized for being black-box models. We tried to stare into this so-called “black box” to identify the variables that drive model performance and understand the extent of these variables’ effects on model performance. In this study, we aimed to generate multiple machine learning models, assess their performance, and select the highest-performing model for clinical practice.

## Materials and methods

### Patient cohorts

This retrospective case‒control study was approved by the institutional review board of the First Affiliated Hospital of Soochow University (Suzhou). Individuals enrolled in our study were treated at Yangzhou Third People’s Hospital (Yangzhou) from August 1st, 2020, to March 31st, 2021. Patients (*n* = 119) confirmed to have COVID-19 by RT‒PCR were included in the case group, presenting with no typical symptoms and no obvious abnormalities in CT images. All positive COVID-19 patients underwent a chest CT exam within 48 h after the RT‒PCR test, and the identified CT scans were reviewed by two experienced radiologists who reached a consensus on the results. Participants in the control group (*n* = 75) were from the health examination population of a hospital from a nonepizootic area; these subjects had two negative RT‒PCR results for COVID-19 within 48 h. Each throat swab was collected at least 24 h apart. Chest CT exams were diagnosed as normal in the control group by two experienced radiologists who reached consensus on the results. The exclusion criteria of the control group included (1) various types of pneumonia (e.g., viral, bacterial and mycoplasma pneumonia), (2) pulmonary tumours, (3) pulmonary emphysema or pneumatocele, (4) tuberculosis, and (5) bronchiectasis.

**Fig. 1:**
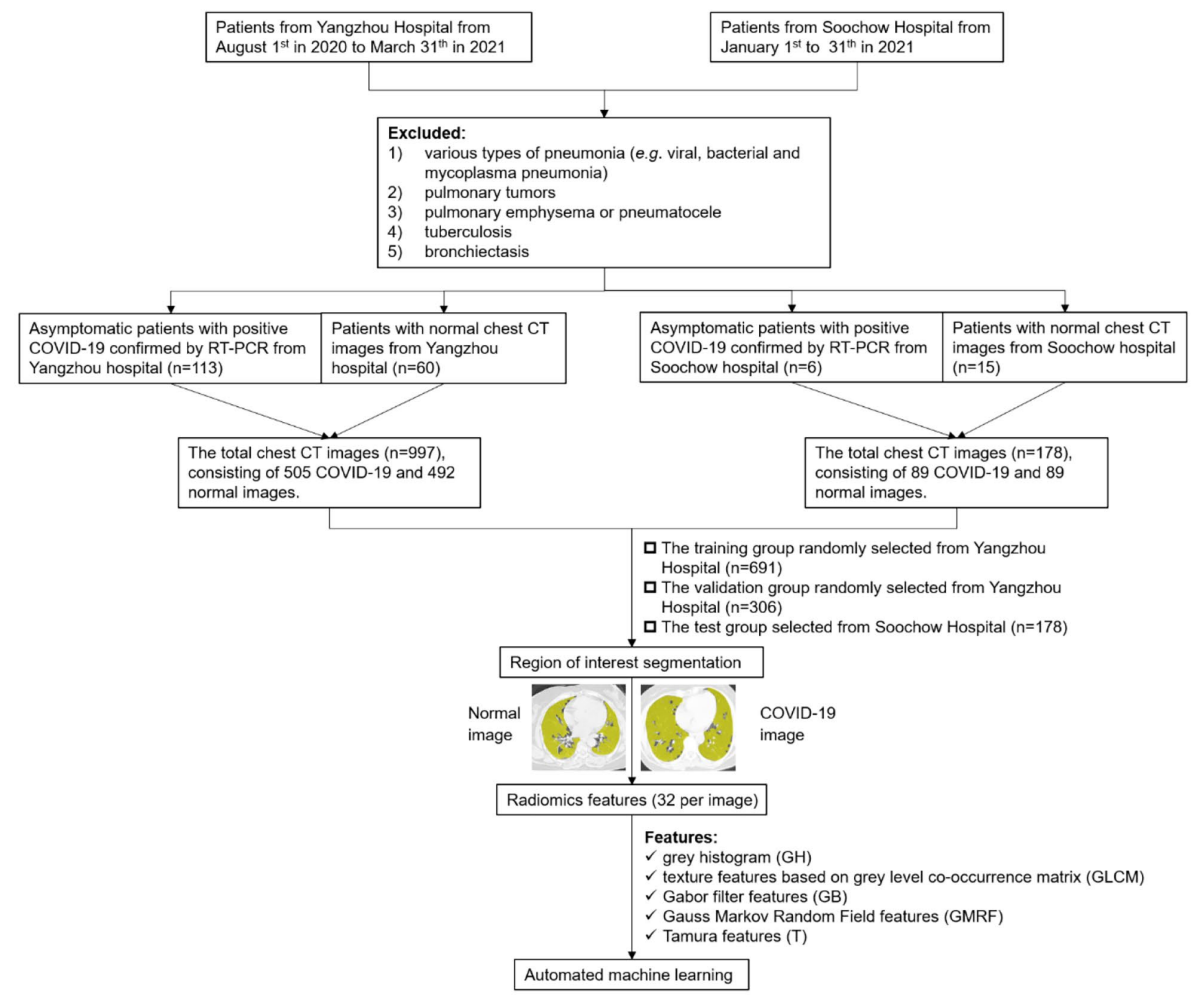
Study flowchart

We randomly split the CT images (*n* = 997) of the aforementioned individuals (*n* = 194) into training (*n* = 691) and internal validation (*n* = 306) datasets to develop the models. Furthermore, these models were tested on CT images (*n* = 178) of individuals enrolled based on the aforementioned inclusion and exclusion criteria from Suzhou from 1st January 2021 to 31st January 2021. The flowchart of our study is shown in Fig. 1.

### Chest CT exams

The identified CT images were directly searched and downloaded from a medical image cloud platform (www.ftimage.cn*).* The lung window was applied to generate 5∼8 images for one individual axial slice in a CT scan with 5 mm thickness, 1500 ± 100 Hounsfield unit (HU) window width and a − 600 ± 50 HU window level. The images were saved in PNG format.

### Image preprocessing

All CT images were pre-processed, and the lung lobes were masked as the region of interest (ROI) using the image processing toolbox in MATLAB (version: R2021b; Natick, MA). We extracted 32 features from each ROI using 5 feature extraction algorithms, including texture features based on a grey histogram (GH) (*n* = 6), texture features based on a grey-level co-occurrence matrix (GLCM) (*n* = 6), Gabor filter features (GB) (*n* = 3), Gauss Markov random field features (GMRF) (*n* = 12) and Tamura features (T) (*n* = 5). Three authors worked together to perform all image segmentations. Three authors independently extracted features from the same set of randomly selected images. To test the differences in image preprocessing between these authors, the Kruskal‒Wallis H test with Dunn post hoc test was used. Furthermore, intraclass correlation coefficient (ICC) analysis was used to calculate the stability between the three authors. Subsequent analysis was continued only when there were no statistically significant differences (*P* > 0.05) in the Kruskal‒Wallis H test and the features had excellent stability (ICC > 0.75).

### Model development and validation based on AutoML

Model development and validation were conducted in R software (version: 4.1.0, The R Foundation) with the H2O package installed from the H2O.ai (cluster version: 3.36.0.2) platform (www.h2o.ai*).* AutoML is a function in H2O that automatically builds a series of machine learning models and finally integrates them into various stacking and ensembled models.

First, the dataset from Yangzhou was randomly split into a ‘training’ (70%) set and a ‘validation’ (30%) set. Second, the training set was used to develop models to predict the probability of COVID-19 infection based on six algorithms, namely, the distributed random forest (RF), random grid of gradient boosting machine (GBM), random grid of deep neural network (DNN), fixed grid of generalized linear model (GLM), random grid of eXtreme gradient boosting (XGBoost) and stacked ensemble (SE) algorithms. Notably, DNN is defined as multilayer perception, a multilayer feedforward artificial neural network containing numerous hidden layers and hyperparameters that works well on tabular data in the H2O official document. The models were then ranked according to their performance on the training set by the AutoML leaderboard. Furthermore, fivefold cross-validation was used to validate these models, and fine-tuned hyperparameters were applied to elevate the performance of the models. The models were developed from the training set based on different algorithms, and the performance of the models was improved by automatically adjusting the hyperparameters and calculating the mean square error (MSE) in the internal validation set. The above process was repeated five times, and then the models with the minimum MSE were obtained. Finally, the performance of the obtained models was verified in a dataset from Suzhou (*n* = 21).

### Statistical analysis

Continuous variables were described as the mean ± standard deviation (SD) if normally distributed or as the median and interquartile range (IQR) if not. The differences in feature extraction among the three authors were compared using the Kruskal‒Wallis H test with Dunn post hoc test. There was no statistical significance when *P* > 0.05, which is representative of feature stability. Image preprocessing and feature extraction were conducted in MATLAB (version: R2021b; Natick, MA), and statistical analysis was performed with R software (version: 4.1.0, The R Foundation) connected with the H2O.ai platform. Data visualization involved a receiver operating characteristic (ROC) curve with an area under the curve (AUC) for model discrimination. Model performance was evaluated based on the AUC, accuracy, sensitivity, specificity, positive predictive value (PPV), negative predictive value (NPV) and F1 score. The F1 score is the harmonic mean of precision and recall. The actual classifications and predictive probabilities were listed as a confusion matrix consisting of true positive (TP), true negative (TN), false positive (FP) and false negative (FN). The formulas are listed as follows: accuracy = $\frac{\text{T}\text{P}+\text{T}\text{N}}{\text{T}\text{P}+\text{F}\text{P}+\text{F}\text{N}+\text{T}\text{N}}$ sensitivity = $\frac{\text{T}\text{P}}{\text{F}\text{N}+\text{T}\text{P}}$ specificity = $\frac{\text{T}\text{N}}{\text{T}\text{N}+\text{F}\text{P}}$ PPV = $\frac{\text{T}\text{P}}{\text{T}\text{P}+\text{F}\text{P}}$ NPV = $\frac{\text{T}\text{N}}{\text{T}\text{N}+\text{F}\text{N}}$ recall = $\frac{\text{T}\text{P}}{\text{T}\text{P}+\text{F}\text{N}}$ precision = $\frac{\text{T}\text{P}}{\text{T}\text{P}+\text{F}\text{P}}$ F1 score = $\frac{2\times \text{p}\text{r}\text{e}\text{c}\text{i}\text{s}\text{i}\text{o}\text{n}\times \text{r}\text{e}\text{c}\text{a}\text{l}\text{l}}{\text{p}\text{r}\text{e}\text{c}\text{i}\text{s}\text{i}\text{o}\text{n}+\text{r}\text{e}\text{c}\text{a}\text{l}\text{l}}$.

## Results

### Feature selection and model optimization using AutoML

A total of 1,175 images were obtained from the COVID-19 group (*n* = 594) and the control group (*n* = 581). High stability with a relatively high intraclass correlation coefficient was shown in image features extracted from these CT images (*P*_*Kruskal−Wallis test*_ > 0.05, Table 1). Six models based on six algorithms were developed, and the performance of the DNN model ranked first among all models, with an AUC value of 0.898 in the test set. As shown in Table 2, all models achieved excellent performance in the training set, with accuracy, sensitivity, specificity, PPV, NPV, F1 score and AUC values beyond 0.990. In the validation set, the AUC value of all models was 1.000, and the SE model obtained the highest accuracy (1.000), followed by the GLM (accuracy = 0.997). Furthermore, the test set results were as follows: DNN model (AUC = 0.898), GLM (AUC = 0.867), SE model (AUC = 0.866), GBM model (AUC = 0.822), RF model (AUC = 0.820) and XGBoost model (AUC = 0.800).

**Table 1:** Differences in image preprocessing among the three authors using the Kruskal‒Wallis H test and ICC analysis

Texture	Features	ICC	95%CI		P _*Kruskal−Wallis H test*_
Grey histogram	Grayscale mean	0.959	0.865	0.985	0.5069
	Contrast mean	0.908	0.779	0.963	0.2446
	R	0.910	0.778	0.964	0.2446
	Third moment	0.961	0.909	0.984	0.6608
	Consistency	0.975	0.921	0.991	0.5838
	Entropy	0.966	0.899	0.988	0.5375
Grey level co-occurrence matrix	Max-probability	0.974	0.913	0.991	0.5871
	Contrast	0.883	0.769	0.948	0.6691
	Correlation	0.822	0.655	0.920	0.4459
	Energy	0.976	0.924	0.991	0.5871
	Homogeneity	0.970	0.903	0.989	0.6326
	Entropy	0.968	0.902	0.988	0.5730
Gabor filter	Texture mean	0.995	0.983	0.998	0.8384
	Contrast	0.994	0.985	0.998	0.8257
	Entropy	0.994	0.980	0.998	0.8777
Gauss Markov random field	GMRF1	0.938	0.875	0.973	0.9784
	GMRF2	0.935	0.862	0.972	0.6458
	GMRF3	0.785	0.611	0.900	0.7857
	GMRF4	0.852	0.716	0.933	0.5980
	GMRF5	0.752	0.560	0.883	0.8573
	GMRF6	0.654	0.420	0.830	0.8505
	GMRF7	0.710	0.501	0.860	0.6711
	GMRF8	0.804	0.639	0.910	0.9584
	GMRF9	0.817	0.661	0.916	0.8218
	GMRF10	0.896	0.798	0.954	0.9863
	GMRF11	0.782	0.605	0.899	0.8561
	GMRF12	0.775	0.592	0.895	0.8077
Tamura	Coarseness	0.991	0.982	0.996	0.9472
	Contrast	0.959	0.895	0.984	0.5367
	Directionality	0.754	0.564	0.884	0.8448
	Line-likeness	0.900	0.788	0.958	0.6008
	Roughness	0.963	0.906	0.986	0.5375

ICC = intraclass correlation coefficient

**Table 2:** Performance of the six models for the three datasets

Models		GBM	XGBoost	GLM	DNN	RF	SE
Training set	AUC	1.000	1.000	1.000	1.000	1.000	1.000
	sensitivity	1.000	1.000	1.000	1.000	1.000	1.000
	specificity	1.000	1.000	1.000	0.994	1.000	1.000
	PPV	1.000	1.000	1.000	0.994	1.000	1.000
	NPV	1.000	1.000	1.000	1.000	1.000	1.000
	accuracy	1.000	1.000	1.000	0.997	1.000	1.000
	F1-score	1.000	1.000	1.000	0.997	1.000	1.000
Validation set	AUC	1.000	1.000	1.000	1.000	1.000	1.000
	sensitivity	0.895	0.993	0.993	1.000	1.000	1.000
	specificity	1.000	0.994	1.000	0.987	0.987	1.000
	PPV	1.000	0.993	1.000	0.987	0.987	1.000
	NPV	0.906	0.994	0.994	1.000	1.000	1.000
	accuracy	0.948	0.993	0.997	0.993	0.993	1.000
	F1-score	0.944	0.993	0.997	0.993	0.993	1.000
Test set	AUC	0.822	0.800	0.867	0.898^*^	0.807	0.866
	sensitivity	0.348	0.742	0.719	0.820	0.809	0.787
	specificity	1.000	0.820	0.978	0.854	0.539	0.910
	PPV	1.000	0.805	0.970	0.849	0.637	0.897
	NPV	0.605	0.760	0.777	0.826	0.738	0.810
	accuracy	0.674	0.781	0.848	0.837	0.674	0.848
	F1-score	0.517	0.772	0.826	0.834	0.713	0.838

DNN, deep neural network, GBM, gradient boost machine; GLM, general linear model; RF, random forest; SE, Stacked ensemble; XGBoost, eXtreme gradient boosting; AUC, area under the receiver operating characteristic curve; PPV, positive predictive value; NPV, negative predictive value; ^*^, the highest AUC value in the test set.

**Fig. 2:**
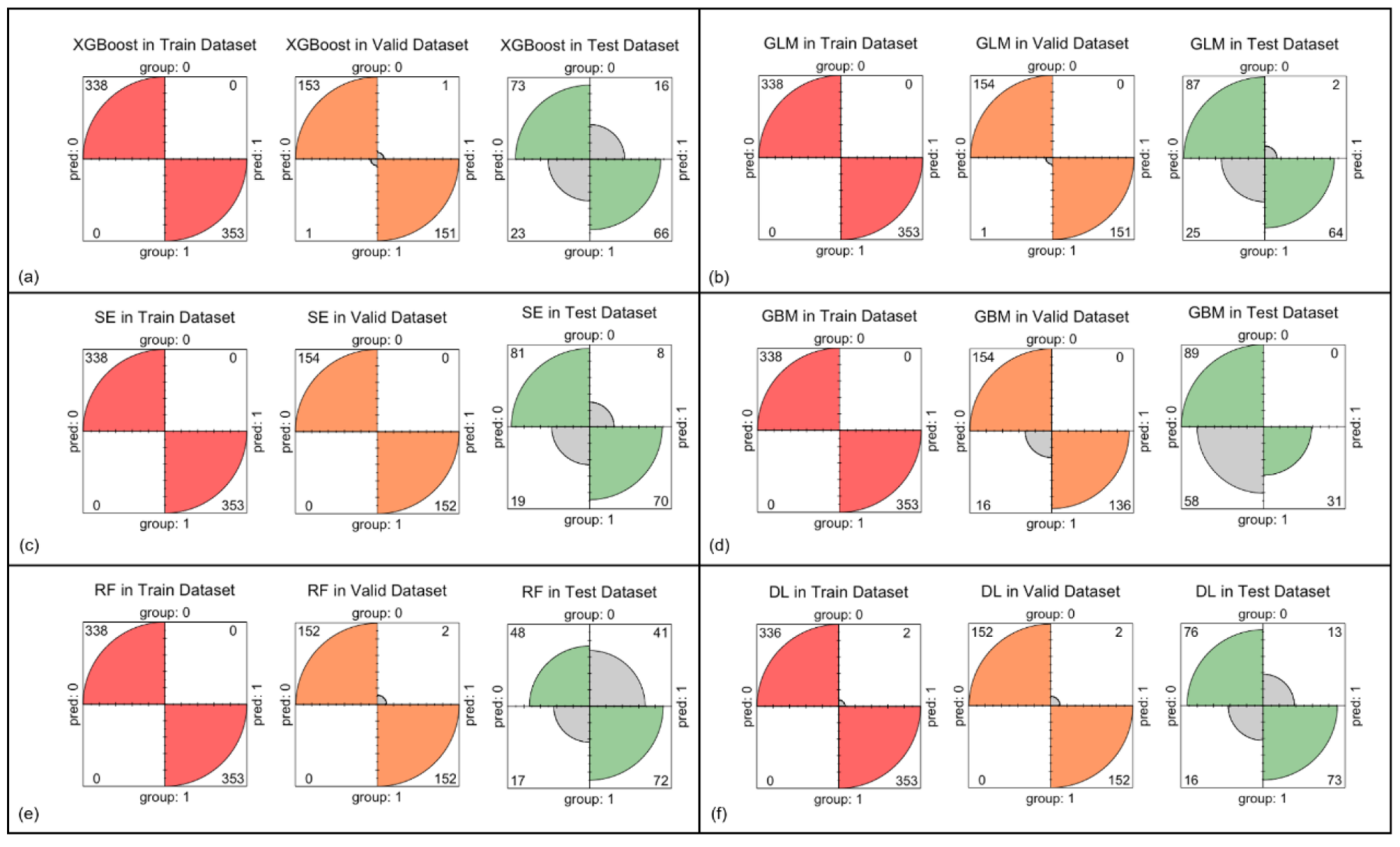
Confusion matrix of the six models for the three datasets

The confusion matrix of the six models in the three datasets is depicted in Fig. 2. False-positive findings in the test set varied by different models, with 17.98% (16/89) for the XGBoost model, 8.99% (8/89) for the SE model, 46.07% (41/89) for the RF model, 2.25% (2/89) for the GLM, 0 (0/89) for the GBM model and 14.61% (13/89) for the DNN model. With regard to true-positive findings, the DNN model detected 73 COVID-19 images among 89 positive images, with the highest sensitivity value of 0.820 for the test set. Other models showed comparable but inferior sensitivity: 0.809 for the RF model, 0.787 for the SE model, 0.742 for XGBoost and 0.719 for the GLM. The GBM model misclassified 58 images among 89 positive images, with the lowest sensitivity of 0.348.

**Fig. 3:**
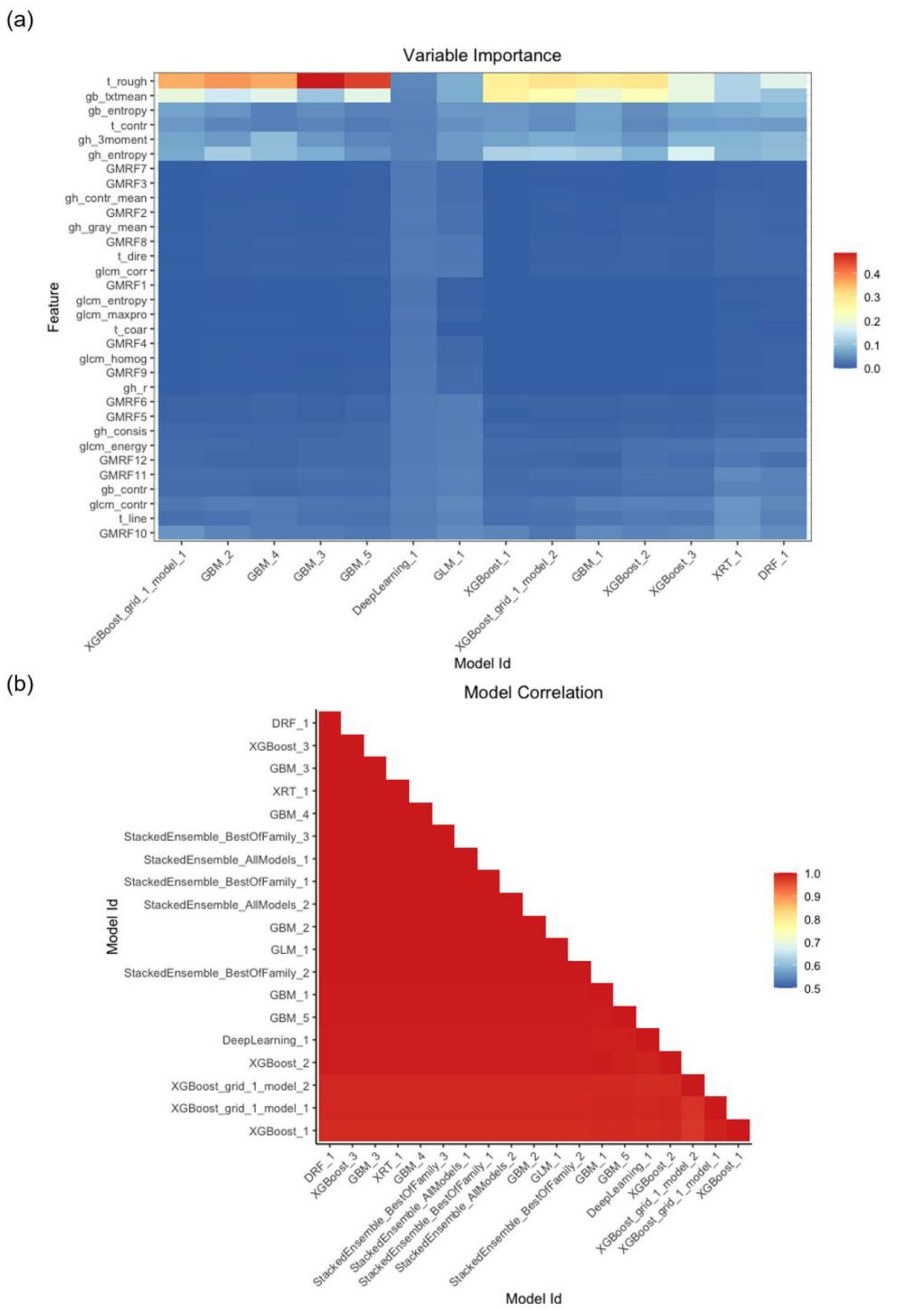
Heatmaps of variable importance (**a**) and model correlation (**b**) based on AutoML in the training set

Heatmaps of variable importance demonstrated the different weights of 32 texture features for different models based on the training set (Fig. 3a). Many models determined that the Tamura roughness was an important variable for predicting the outcome. The models we proposed were highly correlated (Fig. 3b).

### Performance of the best model

As shown in Table 2, the DNN model showed the best ability to distinguish asymptomatic COVID-19 patients from normal controls. The sensitivity, specificity, PPV, NPV, F1 score and accuracy of the DNN model were 0.820, 0.854, 0.849, 0.826, 0.834 and 0.837, respectively (Table 2). To interpret the DNN model, we enumerated several important variables in sequence in Table 3. The texture mean based on GB ranked first, with a relative importance value of 1.000, followed by R based on GB (value = 0.935). Four parameters based on GMRF had values of 0.922, 0.894, 0.833 and 0.818. Correlation based on GLCM and line-likeness based on T ranked fourth and fifth, with values of 0.901 and 0.897, respectively.

**Table 3:** Variable importance rankings for the best AutoML model algorithm (DNN)

Variables ID	Ranking	Relative importance	Variables
GB_txtmean	1	1.000	Texture mean based on Gabor filter
GB_R	2	0.935	R based on Gabor filter
GMRF6	3	0.922	6th parameter of Gauss Markov random field
GLCM_corr	4	0.901	Correlation based on grey-level co-occurrence matrix
T_line	5	0.897	Line-likeness based on Tamura algorithm
GMRF11	6	0.894	11th parameter of Gauss Markov random field
GMRF12	7	0.833	12th parameter of Gauss Markov random field
GMRF7	8	0.818	7th parameter of Gauss Markov random field

**Fig. 4:**
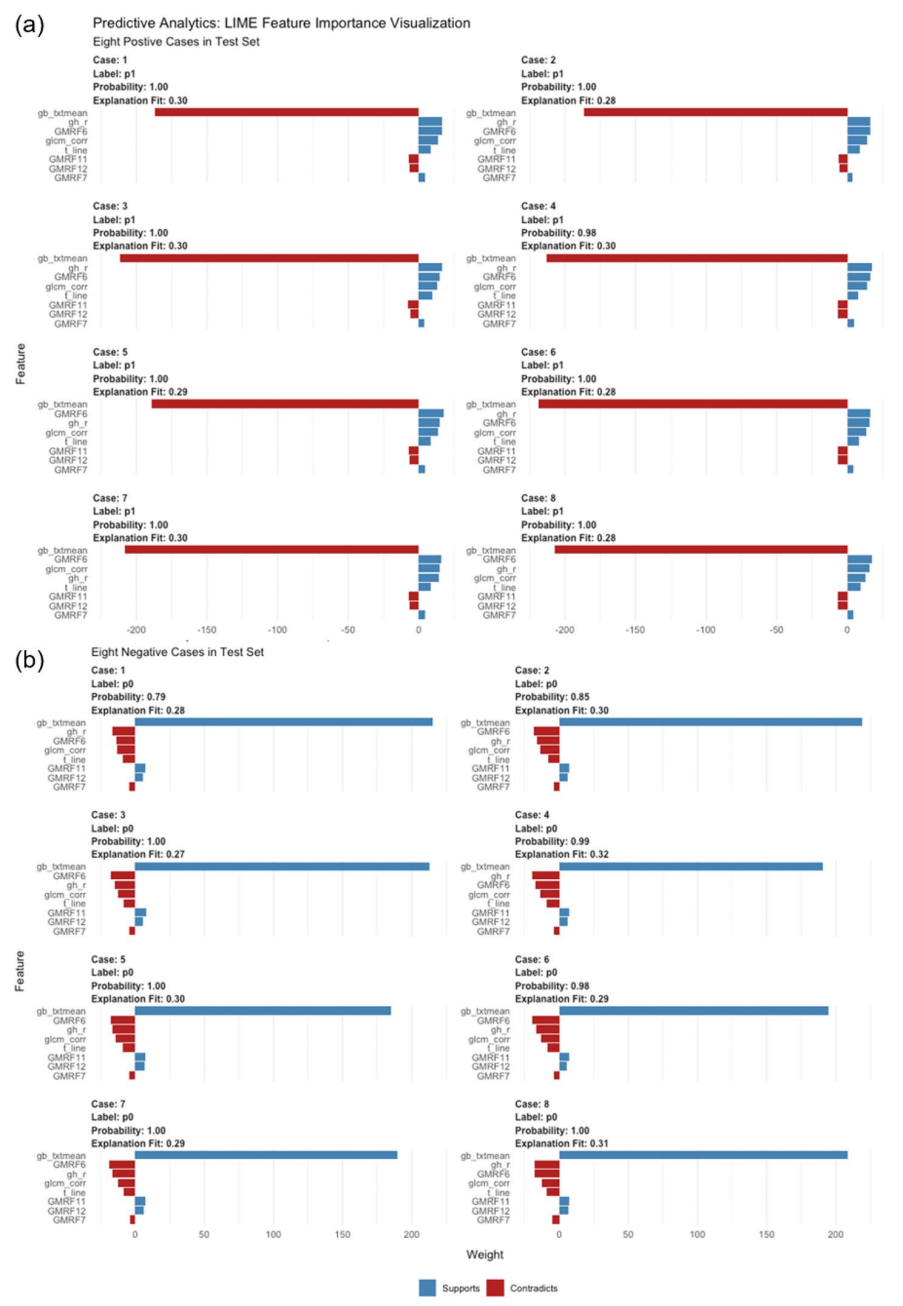
Local interpretable model-agnostic explanation (LIME) of the deep learning model in the test set. (a) shows how eight key features contributed to predicting positivity for the eight COVID-19 cases. (b) shows how eight key features contributed to predicting negative results for the eight normal cases

A plot of local interpretable model-agnostic explanation (LIME) demonstrated how different variables work in separating the asymptomatic from the normal. The red contradicted the prediction, while the blue supported the prediction. As shown in Fig. 4a, positive case 1 was predicted to be asymptomatic, with a probability of 1.00. The texture mean based on GB contributed the most to the prediction, followed by R based on GH. Other cases were explained and are shown in Fig. 4. Additionally, negative case 1 in Fig. 4b was judged as normal by the DNN model, with a probability of 0.79. The texture mean based on GB also had the highest weight based on the DNN model.

## Discussion

### Principal findings

We used AutoML to successfully generate multiple machine learning models, assess their performance, and select the highest-performing models for predicting asymptomatic surviving COVID-19 infection. Our study demonstrates that machine learning models that use CT image characteristics can identify asymptomatic patients. Clinicians can just type in the image omics features and get a prediction probability. Positive nucleic acid result was hard to get just from one or twice throat swab samples. If some patients was suspected with COVID-19 carriers, but with no typical symptom, no typical CT viral pneumonia imaging performance, this AI model we built could help identify these asymptomatic patients, or could provide evidence for clinician to get the deeper airway samples like tracheoscopic perfusion.

AI, including machine learning and deep learning, has been widely used in medical fields such as disease diagnosis [14], lesion detection [15], and prognostic analysis [16]. Previous studies revealed the potation of AI in medical imaging [17–18]. A systematic review summarized a total of 48 studies about AI methods applied to COVID-19 diagnosis, biomarker discovery, therapeutic evaluation and survival analysis from January 2020 to June 2022 [19]. This review provided evidence to delineate the potential of AI in analysing complex gene information for COVID-19 modeling on multiple aspects including diagnosis. These gene information is very significant. Baktash et al. trained an ensemble bagged tree model using clinical parameters but not CT scan for detecting atypical COVID-19 presentations with an accuracy of 81.79%, sensitivity of 85.85% and specificity of 76.65% [20]. These studies showed that AI has the potential to diagnose the COVID-19. Yan et al. retrospectively collected 206 patients with positive RT-PCR for COVID-19 and their chest CT scans with abnormal findings, and results showed that the CNN model was able to differentiate COVID-19 from other common pneumonias based on the CT scan level [21]. His study showed that machine learning just using CT scam might identify the COVID-19. Thus, we developed a series of deep learning models to identify asymptomatic COVID-19 patients based on CT images, which achieved good performance with accuracy values ranging from 0.933 to 0.980 in the test set [22]. This published study by our team showed that machine learning showed high accuracy in diagnosing asymptomatic COVID-19 patients. However, previous deep learning model is a kind of black box model, where we don’t know how deep learning frameworks recognize these two kinds of CT images. In this study, we used the image omics code to extract interpretable features, such as shape features, first-order statistical features, gray scale co-occurrence matrix and so on. Based on these features, the machine learning model classified CT images into two categories (COVID-19 and non- COVID-19). Our study used machine learning algorithms to differentiate asymptomatic patients from normal subjects based on CT images and achieved high accuracy, indicating that AI is an efficient and informative tool for medical systems and promotes better decision-making.

The advantage of AutoML is that it is not limited to dealing with numerous medical data by powerful computational capability; it can also reduce time-consuming costs and labour-consuming costs. Uthman et al. developed five AI classifiers to predict whether a study was eligible for their systematic analysis of complex interventions using AutoML, indicating that the best classifier yielded a workload saving of 92% [23]. Zhang et al. compared four AutoML frameworks, AutoGluon, TPOT, H2O and AutoKeras, that performed better than traditional machine learning algorithms, such as support vector machine algorithms [24]. The authors indicated that AutoML could reduce the time and effort devoted by researchers due to its automatic model optimization. In our study, AutoML code was introduced from the open-access H2O.ai platform. The process of parameter tuning and optimal algorithm selection was automatic, and we set the running time of AutoML to 30 s. The promising results demonstrated that AutoML is time-efficient and labour-saving with comparable predictive performance.

Radiomic medicine can extract a large amount of texture feature information from images to reflect the heterogeneity of damage. For example, GH is a first-order statistical feature that depicts the distribution of grey-level intensities [25]. The GLCM mainly reflects the characteristics of the internal structure of the image through the change in density [26–28]. Filters can display the spatial heterogeneity of tumours using wavelet transformation [16]. GMRF is used to remove inconsistency in the pixel level of slide images [29]. Therefore, even if no lesions are found on the CT images, we can analyse different types of texture features extracted to determine whether the lung tissue is damaged. Our results showed that the best model was the DNN model. The XGBoost model, SE model, RF model, GLM, and GBM model performed slightly worse than the DNN model. We used the AUC as our metric of model utility because it accounts for model sensitivity and specificity. According to the DNN model, the texture mean based on GB ranks first in importance. R based on the Gabor filter, the 6th parameter of GMRF, correlation based on GLCM, line-likeness based on the Tamura algorithm, the 11th parameter of GMRF, the 12th parameter of GMRF and the 7th parameter of GMRF ranked in sequence among CT characteristics using the DNN model. Our results showed that these CT characteristics occupied a decisive position in distinguishing asymptomatic carriers.

The diagnosis of asymptomatic COVID-19 carriers is difficult due to no abnormal pathological changes in the lung in radiological images and no apparent symptoms, such as fever, cough and expectoration [2, 30]. A comprehensive review summarized currently available AI devices to monitor and detect asymptomatic COVID-19 carriers early using vital data [31]. Ozturk et al. differentiated normal from COVID-19-infected subjects using deep learning (DL) models, achieving an average accuracy of 98.08% based on X-rays [15]. Yasar et al. [32] developed machine learning (ML)-based and DL-based classifiers to distinguish between COVID-19 and non-COVID-19 on CT images, with over 0.9197 AUC values under 2- and 10-fold cross validation. This study used the AutoML method based on CT radiomic features to study asymptomatic COVID-19 patients to find changes in nonfocus areas that humans cannot find. These models also had high sensitivity values, specificity values, and NPVs.

### Clinical insights into the Black Box

The trade-off between predictive power and interpretability is a common issue when working with black-box models, especially in medical environments where results have to be explained to medical providers and patients. Interpretability is crucial for questioning, understanding, and trusting AI and machine learning systems.

According to our variable importance heatmap, many models determined that the Tamura roughness exhibited substantial weight for predicting the outcome. The Gabor filter-texture mean was also an influential variable. The confusion matrix of six models for the three datasets provided insight into the black box. The GBM model presented the highest specificity. The DNN model presented the highest sensitivity. The LIME plot of the DNN model allowed us to determine the importance of variables and provided information on how the variables influenced the models’ predictions. It provided numerical information on variables’ effects. For example, the LIME showed that the GB-Texture mean was associated with an increased probability of negative and a decreased probability of positive results. The large weight ratio of GB-Texture to predict the result supports the idea that CT with low GB-Texture indicates an increased risk of infection. Further exploration is needed to confirm clinical findings and show clinical thresholds.

### Limitations

Firstly, a total of 1,175 images from 173 cases were included in our study; thus, the sample number was relatively insufficient. Further exploration in more cities was needed. Secondly, there was no complete biological explanation of the radiomic features in this study, and further exploration is needed in the future. Thirdly, the best DNN model achieved the highest AUC and F1-score, but the specificity and PPV were lower than those of the GLM and SE models. This result indicated that there might be misdiagnosis if the DNN model is used in clinical practice. Fourthly, demographics of the participants was not analysed in this study. Whether the difference existed among the participants was not sure. This is the limitation for broader application. Lastly, manual image preprocessing was conducted before AutoML analysis, which was time- and labour-consuming. Despite the high consistency of image preprocessing, the heterogeneity of devices from different institutions is still inevitable.

## Conclusion

In conclusion, we believe that AutoML models based on radiomic features of chest CT images can effectively classify asymptomatic COVID-19 carriers. In the future, we plan to continue research in three areas: first, deep radiomics, which can automatically segment the lung lobes and extract radiomic features using novel technologies, i.e., transfer learning. In addition, augmenting dataset samples from multiple centres is helpful to further ensure model generalization and robustness. Prospective experiments also need to be considered to evaluate model reliability in clinical decisions. Furthermore, we should investigate the association between radiomic features and biological significance to explore new mechanisms to improve our model.

## Data Availability

The datasets used during the current study are available from the corresponding author upon reasonable request.

## Abbreviations

AI: Artificial intelligence
AUC: Area under receiver operating characteristic curve
AutoML: Automated machine learning
COVID-19: Coronavirus disease 2019
CT: Computed tomography
DNN: Deep neural network
FN: False negative
FP: False positive
GB: Gabor filter features
GBMs: Gradient boosting machines
GGO: Ground-glass opacity
GH: Grey histogram
GLCM: Grey-level co-occurrence matrix
GLMs: Generalized linear models
GMRF: Gauss Markov random field features
HU: Hounsfield unit
ICC: Intraclass correlation coefficient analysis
IQR: Interquartile range
LIME: Local interpretable model agnostic explanation
MSE: Mean square error
NPV: Negative predictive value
PPV: Positive predictive value
RF: Distributed random forest
ROC: Receiver operating characteristic
RT‒PCR: Reverse-transcription polymerase chain reaction
SD: Standard deviation
SE: Stacked ensemble
Suzhou: The First Affiliated Hospital of Soochow University
T: Tamura features
TN: True negative
TP: True positive
WHO: World Health Organization
XGBoost: EXtreme gradient boosting
Yangzhou: Yangzhou Third People’s Hospital
